# Enhancement of InN Luminescence by Introduction of Graphene Interlayer

**DOI:** 10.3390/nano9030417

**Published:** 2019-03-12

**Authors:** Darius Dobrovolskas, Shingo Arakawa, Shinichiro Mouri, Tsutomu Araki, Yasushi Nanishi, Jūras Mickevičius, Gintautas Tamulaitis

**Affiliations:** 1 Institute of Photonics and Nanotechnology, Vilnius University, Sauletekio al. 3, LT-10257 Vilnius, Lithuania; juras.mickevicius@ff.vu.lt (J.M.); gintautas.tamulaitis@ff.vu.lt (G.T.); 2 Department of Electrical and Electronic Engineering, Ritsumeikan University, Kusatsu, Shiga 525-8577, Japan; re0052ks@ed.ritsumei.ac.jp (S.A.); iguchan@fc.ritsumei.ac.jp (S.M.); tara@se.ritsumei.ac.jp (T.A.); nanishi@se.ritsumei.ac.jp (Y.N.)

**Keywords:** graphene, indium nitride, molecular beam epitaxy, photoluminescence

## Abstract

Indium nitride (InN) luminescence is substantially enhanced by the introduction of a multilayer graphene interlayer, mitigating the lattice mismatch between the InN epilayer and the Gallium nitride (GaN) template on a sapphire substrate via weak van der Waals interaction between graphene and nitride layers. The InN epilayers are deposited by radio-frequency plasma-assisted molecular beam epitaxy (MBE), and are characterized by spatially-resolved photoluminescence spectroscopy using confocal microscopy. A small blue shift of the emission band from the band gap evidences a low density of equilibrium carriers, and a high quality of InN on multilayer graphene. A deposition temperature of ~375 °C is determined as optimal. The granularity, which is observed for the InN epilayers deposited on multilayer graphene, is shown to be eliminated, and the emission intensity is further enhanced by the introduction of an aluminum nitride (AlN) buffer layer between graphene and InN.

## 1. Introduction

Indium nitride (InN) is a promising material for near-infrared optoelectronic devices owing to its narrow band gap (~0.65 eV), small electron effective mass, and high peak electron velocity [1]. However, extensive implementation of this semiconductor is hindered by low crystal quality of InN epitaxial layers, which are deposited on foreign substrates, since the native substrates for InN are unavailable [2,3,4].

In principle, the low emission efficiency caused by the low crystal quality can be circumvented by controlling energy transfer between radiating dipole and localized plasmons [5]. However, it does require careful adjustment of metal nanoparticle size to match plasmons resonance with emission peak. A more straightforward approach is to decrease the nonradiative recombination rate by improving the crystal quality itself. Van der Waals epitaxy (vdWE) was recently suggested as a prospective approach to mitigate the lattice mismatch in growing heteroepitaxial layers of III-nitrides [6]. A distinctive feature of the vdWE approach is utilizing the weak van der Waals interaction between the epitaxial layer and an intentionally introduced interlayer, instead of the strong chemical bonds exploited by conventional epitaxy [7]. Graphene is a prospective candidate to be used as a relieving vdWE layer to subsequent layers of III-nitrides. Since graphene has a hexagonal symmetry, it is effective for facilitating epitaxial growth of c-plane of GaN, even on substrates without a hexagonal sixfold symmetry [8].

This approach opens a new route to alleviate one of the most critical problems in III-nitride growth, i.e., the substrate compatibility. In future perspective, combining distinctive band structure and physical properties of graphene with III-nitrides may lead to novel optoelectronic devices.

It is already demonstrated that the introduction of graphene improves the crystal quality and luminescence intensity of GaN [8,9], AlN [10], and Indium gallium nitride (InGaN) [6,11]. The improvement of structural quality is demonstrated also for InN on graphene [12]; however, no study of the luminescence of InN on graphene is still reported. In this study, we show that the photoluminescence (PL) properties of InN on multilayer (ML) graphene are substantially better than those of InN without ML graphene interlayer, and might be improved by an appropriate selection of deposition conditions. To perform InN epitaxy, exfoliated ML graphene was exploited in the current work, because of the high quality of this type of graphene. Coupling of our photoluminescence spectrometry system with a confocal microscope enabled us to study the homogeneity of InN epilayers, and compare the emission from areas with and without the graphene interlayer.

The InN deposition has been accomplished by radio-frequency plasma-assisted molecular beam epitaxy (rf-MBE), which is known to provide a better structural quality of InN epilayers than that achievable by metalorganic chemical vapor deposition (MOCVD). The deposition temperatures were varied, and the optimal growth temperature was estimated. Additionally, a sample with an AlN interlayer, introduced between ML graphene and InN to affect the coalescence processes in the InN layer deposition, was prepared and studied.

## 2. Materials and Methods

Exfoliation of graphite was used to prepare multilayer (ML) graphene flakes which were then transferred onto gallium nitride (GaN)-on-sapphire templates. Indium nitride (InN) layers were deposited using radio-frequency plasma-assisted molecular beam epitaxy (rf-MBE), setup (EpiQuest RC2100NR, Kyoto, Japan). We used droplet elimination by radical ion beam (DERI) process [13] for the growth. Indium supply period (5 min) and nitrogen-rich supply period (3–5 min), have been repeated twice. The period of nitrogen-rich process was determined by the reflection high-energy electron diffraction (RHEED) monitoring. To optimize the growth conditions, a set of samples was grown at different temperatures: 325, 375, 425, and 435 °C (samples S325, S375, S425, and S435, respectively). An additional sample (A425) having an aluminum nitride (AlN) buffer layer between InN and ML graphene, was prepared at 425 °C to study the possible effect of AlN layer on coalescence in InN layer. The growth temperature was calibrated using a pyrometer.

The surface topography was analyzed with a 10-nm lateral resolution, using atomic force microscopy (AFM) in tapping mode (Bruker Dimension Icon, Billerica, MA, USA). The crystal structure of InN layers was studied by the electron backscatter diffraction (EBSD) technique. The emission properties were studied by photoluminescence (PL) spectroscopy using a confocal microscope (WITec Alpha 300, Ulm, Germany). A CW laser diode emitting at 660 nm (Integrated Optics) was used as the excitation source. The PL spectra were recorded using a spectrometer (Andor Shamrock, Belfast, UK) equipped with an Indium gallium arsenide (InGaAs) detector array (Andor, Belfast, UK). The spatial resolution of 1.3 µm was achieved using a high numerical aperture (NA = 0.55) microscope objective. To eliminate the noise from the confocal microscopy InGaAs images, PL spectra were approximated by a Gaussian function at every pixel of the image before extracting PL parameters. All the measurements were performed at room temperature.

## 3. Results and Discussion

**Figure 1:**
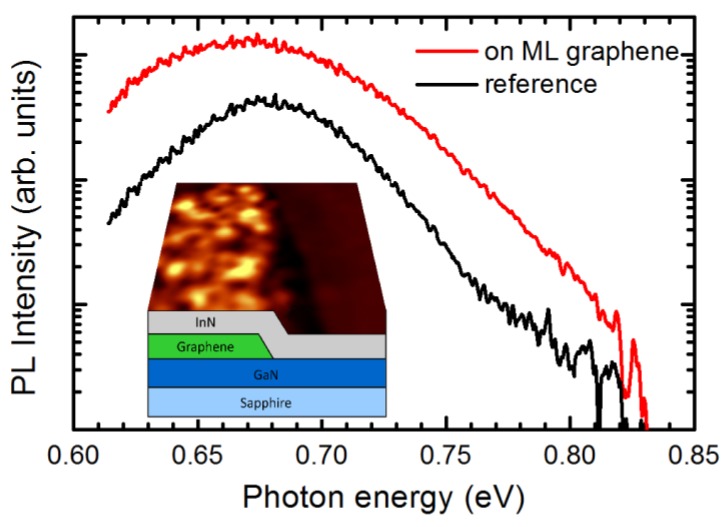
Spatially-integrated photoluminescence (PL) spectra from Indium nitride (InN) areas deposited on multilayer (ML) graphene and without ML graphene interlayer of sample S375 grown at 375 °C. The inset schematically illustrates the sample structure (not to scale).

Typical spatially-integrated PL spectra from InN deposited on ML graphene flakes (hereafter, InN on ML graphene) and InN layer outside ML graphene (hereafter, reference area) are shown in Figure 1. The inset in Figure 1 schematically illustrates the sample structure. The PL band of the InN on ML graphene peaks at 0.669 eV, and exhibits no significant shifting in all layers under study deposited at different temperatures. This long-wavelength position close to the band gap of InN, 0.65 eV [14], evidences a weak conduction band filling effect, which is usually stronger in InN due to a high density of equilibrium electrons released into the conduction band from defect-related donors.

The most long-wavelength position of the PL band of MBE-grown InN was observed at 0.63 eV [14] and 0.64 eV [15], while the PL band in InN epilayers grown by metalorganic chemical vapor deposition (MOCVD) peaks at a shorter wavelength, 0.70 eV [16] or 0.72 eV [17]. The most important feature is the enhancement of the PL emission by a factor of three in the InN layers deposited on ML graphene interlayer.

**Figure 2:**
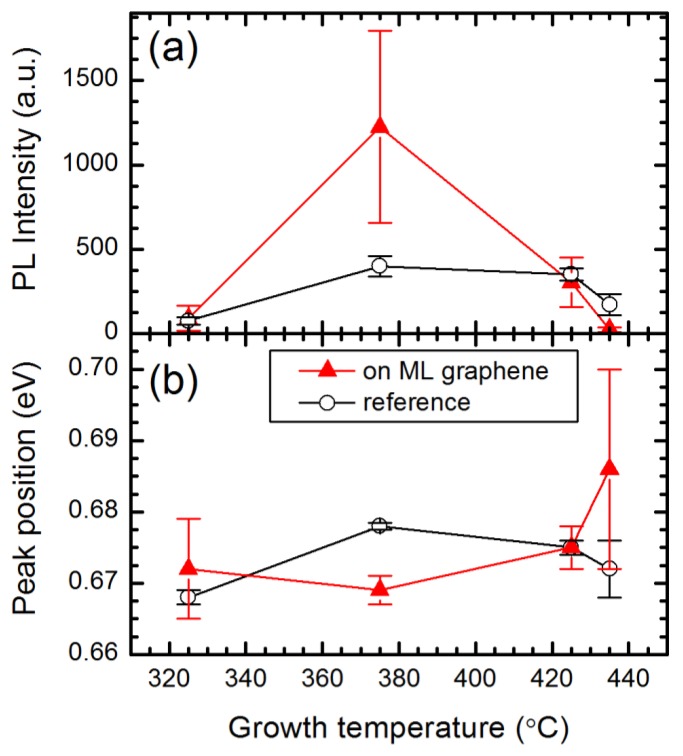
Average PL intensity (**a**) and peak position (**b**) of InN layers grown at different temperatures. PL parameters from the areas on ML graphene (red triangles) and outside ML graphene (open circles) are compared.

To maximize the positive effect of the ML graphene interlayer, the InN deposition temperature was varied. The effect of growth temperature on PL parameters is demonstrated in Figure 2. Error bars indicate standard deviations. For each sample, the PL parameters were spatially averaged from InN on ML graphene areas and from the reference areas without graphene. The PL intensity increases as the growth temperature is increased from 325 °C to 375 °C, and decreases at elevated temperatures. The deterioration of luminescence properties even at the temperatures as low as 425 °C is probably caused by the enhanced dissociation of InN on ML graphene because of weak van der Waals interaction. As a result, the optimal temperature for InN deposition on ML graphene is in the vicinity of 375 °C. Moreover, sample S375 exhibits the lowest in the sample set PL peak position of InN on ML graphene (0.669 eV). This is an indication that this temperature ensures deposition of InN on ML graphene epilayers with the lowest density of residual electrons, and consequently, the weakest band filling effect.

**Figure 3:**
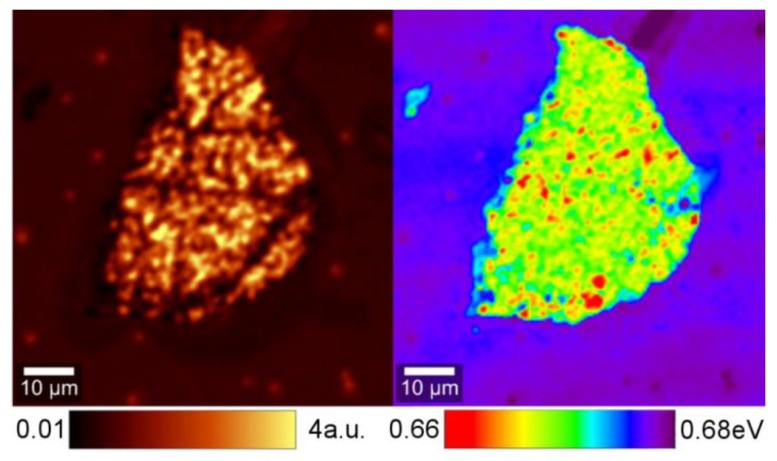
PL peak intensity (**left**) and PL band peak position (**right**) mapping images of InN layer grown on a ML graphene flake at 375 °C temperature.

Typical images of the spatial distribution of PL peak intensity and band peak position of the samples studied are shown in Figure 3. The ML graphene flakes exploited for the deposition had irregular shapes with the characteristic dimensions of a few tens of micrometers. The PL intensity of the InN layers deposited on these flakes is spatially inhomogeneous. The spatial PL intensity variation (the standard deviation divided by the average PL intensity value) in InN on ML graphene depends on the deposition temperature: It decreases from 0.82 in sample S325 to 0.46 in S375 and increases again up to 0.53 in S435. Meanwhile, the intensity variation in the reference areas without the ML graphene interlayer is considerably smaller: This decreases from 0.29 in sample S325 to 0.10 in S425 and increases to 0.35 in S435.

**Figure 4:**
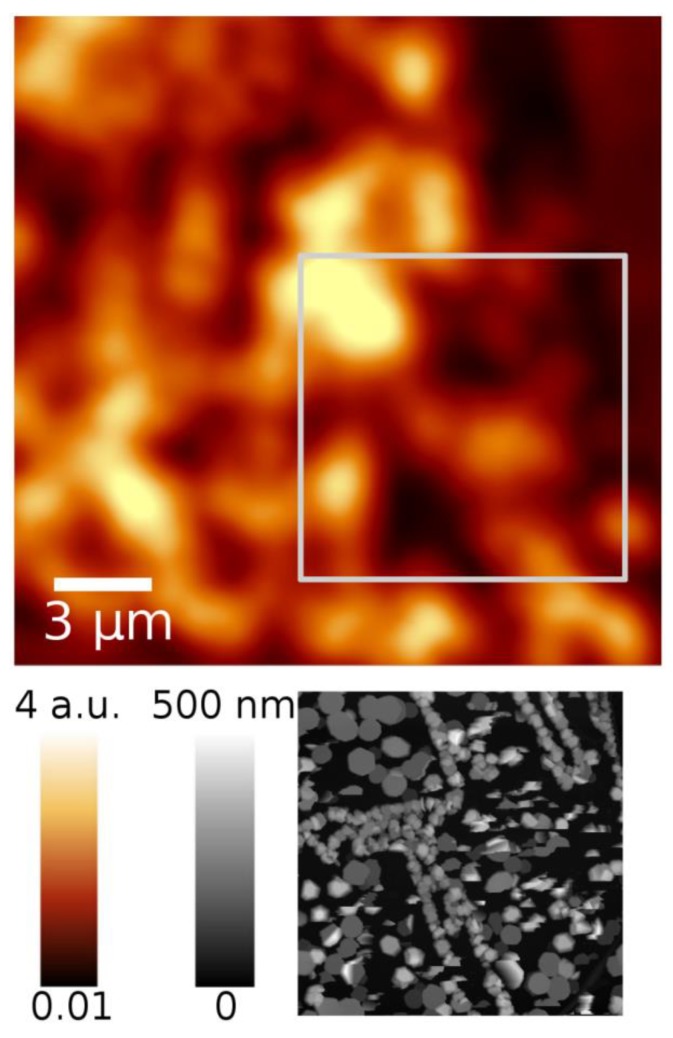
PL intensity image (red-yellow) and an atomic force microscopy (AFM) image (grayscale) of the square indicated of the InN sample grown on ML graphene at 375 °C.

To compare the spatial inhomogeneity of PL parameters with surface morphology, the PL images obtained using the confocal microscope were matched with atomic force microscopy (AFM) images. A combined image for sample S375 is shown in Figure 4. The PL intensity distribution contains bright areas with typical dimensions of ~1.5 μm on a darker background. As seen in AFM images, the bright areas, as well as the dark ones, are not structurally homogeneous and contain several InN grains with planar sizes of ~0.2 µm, and an average height of ~0.14 µm. The grain size depends on deposition temperature. In general, the variation in grain planar size and height of InN on ML graphene is substantially larger than that of InN directly on GaN template. The root mean square (RMS) values of InN grain height are 73, 121, 95, and 121 nm on ML graphene, while 0.6, 2.0, 2.6, and 15 nm on the areas without ML graphene sublayer, for samples S325, S375, S425, and S435, respectively.

Meanwhile, the average lateral size of InN grains on ML graphene increases from 125 nm in InN deposited at 325 °C to 256 nm at 375 °C, and decreases to 192 and 186 nm as the temperature is further increased to 425 °C and 435 °C, respectively. This grain size variation might be explained by a low migration barrier of group-III metals on ML graphene due to weak van der Waals interaction between InN and graphene, and by the dissociation of InN reducing the grain size at elevated temperatures [18].

**Figure 5:**
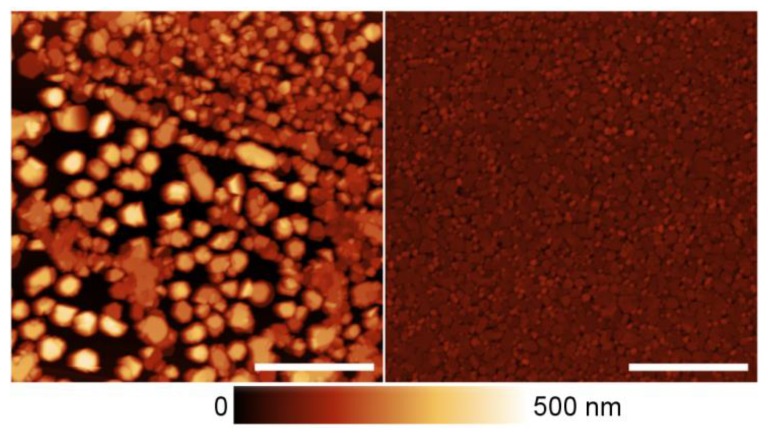
AFM images of InN layer deposited at 425 °C on ML graphene without (**left**) and with an aluminum nitride (AlN) interlayer (**right**). White scale bar is 3 µm.

**Figure 6:**
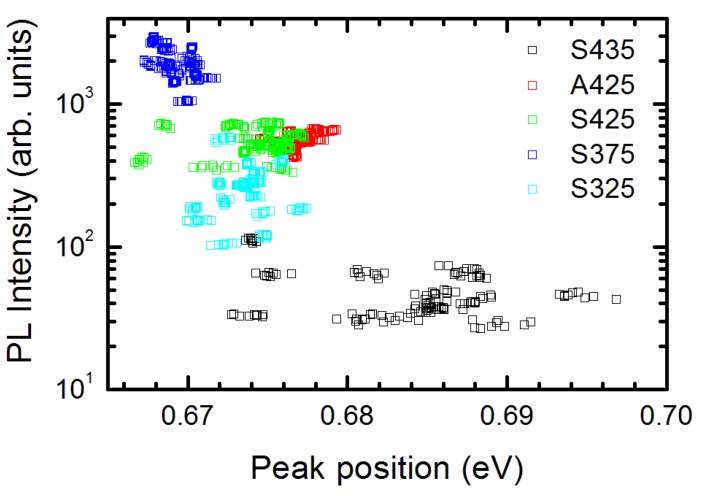
PL intensities and band peak positions in bright spots on different samples (color-coded, numbers indicated) of InN on ML graphene.

We attempted to improve the homogeneity of InN epilayers on ML graphene by introducing additional interlayers. The lack of dangling bonds on the surface of pristine graphene obstructs the direct growth of III-nitrides, as there are no sites to promote bonding with foreign atoms. To overcome this issue, the graphene surface can be functionalized. Buffer layers of AlN [12,19] and ZnO [20,21] on graphene were previously utilized to facilitate the growth of GaN layers or LED structures. In the current work, the effect of a buffer layer was investigated by growing a thin AlN layer between the ML graphene interlayer and InN epilayer deposited at 425 °C (sample A425). Typical AFM images are shown in Figure 5. AlN layer substantially improves the homogeneity of InN layer on ML graphene. The RMS of InN layer on ML graphene decreases from 94.9 nm (sample S425) to 17.4 nm (A425). However, the improved structural homogeneity of the InN layer did not improve the luminescence properties. The comparison of the results provided in Figure 6 for sample S425 deposited at 425 °C, and sample A425 deposited at the same temperature, but with an AlN interlayer serving for substantial structural homogenization of the layer, shows that the homogenization does not result in the higher efficiency of the bright spots. Thus, the observed increase by 56% in spatially-integrated PL intensity by introduction of AlN interlayer is caused by higher surface filling with closely-packed InN grains rather than by luminescence efficiency.

Moreover, the average PL peak position is slightly blueshifted by the introduction of an AlN interlayer. This is an indication of a higher density of donor states.

The smallest observed diameter of the bright spots in PL peak intensity images is limited to ~1.3 µm by the spatial resolution of the confocal microscope. Nevertheless, the comparison of the images with the AFM images leads to a conclusion that the overall PL efficiency is higher in the areas containing larger InN grains. The overall PL efficiency, considered here as the ratio of the numbers of photons emitted per photons incident, depends on (i) excitation conditions, (ii) internal quantum efficiency, and (iii) light extraction efficiency. A dedicated study is necessary to reveal the relative contributions of these three efficiencies.

A high density of unintentional donors and, consequently, strong filling of the conduction band resulting in a high-energy shift of the optical band gap is an inherent property of InN. Therefore, we studied the correlation of PL intensity and band peak position. In each sample, we selected regions of 0.6 × 0.6 µm² in size at the center of several bright spots, and extracted the PL intensity and the band peak position spatially-integrated within the regions. The pairs of the PL intensity/peak position values for each region in the five samples studied are represented as points in Figure 6. The results reveal a general trend: The samples exhibiting lower-energy PL peak position emits at a higher efficiency. Moreover, the higher is the peak position, the larger are the distributions of the PL peak position, the band width, and the emission intensity in the bright spots of the InN layers.

**Figure 7:**
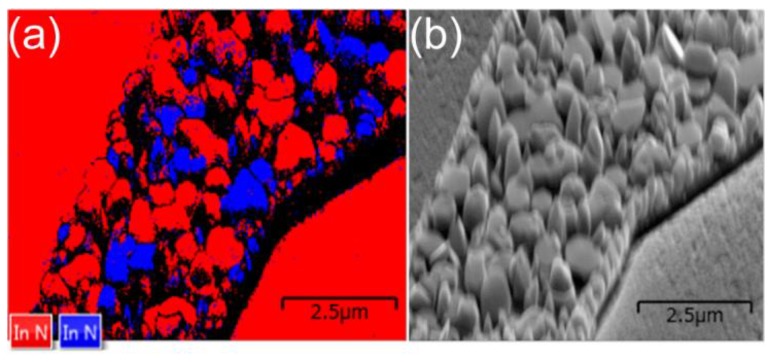
Typical EBSD (**a**) and SEM (**b**) images of InN on ML graphene flake. The hexagonal and cubic phases of InN are presented in red and blue, respectively.

The large PL intensity and band width variations might be explained by the mixing of hexagonal and cubic crystal phases on ML graphene flakes. The contribution of cubic and hexagonal phases was previously suggested to explain double-peaked PL spectra of GaN on graphene [8]. In InN, the hexagonal and cubic phases cannot be identified using only PL parameters, since the PL bands of hexagonal and cubic InN strongly overlap [22]. However, the mixing of hexagonal and cubic phases of InN on ML graphene flakes was confirmed by electron backscatter diffraction (EBSD). A typical EBSD image of InN on ML graphene flake is presented in Figure 7a. The hexagonal and cubic phases of InN are presented in red and blue, respectively. A Scanning Electron Microscope (SEM) image of the same area is presented in Figure 7b, for comparison. Note that InN deposited on graphene-free areas predominantly consists of hexagonal phase. Therefore, the PL peak position and band width variations there are smaller in these areas.

## 4. Conclusions

In conclusion, InN epilayers were grown by rf-MBE on exfoliated ML graphene transferred onto GaN-on-sapphire templates. Substantial enhancement of PL intensity of InN by introduction of ML graphene interlayer is observed. The introduction of an ML graphene interlayer also results in a smaller blue shift of the PL band in respect to the band gap of InN, which indicates a lower density of donors providing free electrons into the conduction band. The optimal growth temperature for the deposition of InN on ML graphene was found to be in the vicinity of 375 °C. It is shown that the InN epilayers on ML graphene are inhomogeneous and consist of grains ranging from 0.2 to 1.2 µm in size. An AlN buffer layer was found to improve the homogeneity of the InN layer and to further enhance the spatially-integrated PL intensity. The comparison of the spatially-resolved PL measurements with the surface morphology characterized by AFM shows that the enhancement is caused by surface filling rather than by the enhancement of PL efficiency. The results demonstrate that the ML graphene interlayers are beneficial for growing thin epitaxial layers of InN with high overall emission efficiency. Further optimization of growth conditions to enhance the overall emission efficiency by improving the surface coverage, light extraction conditions and internal quantum efficiency are feasible.
